# Fundamental study on quality assurance (QA) procedures for a real‐time tumor tracking radiotherapy (RTRT) system from the viewpoint of imaging devices

**DOI:** 10.1002/acm2.13307

**Published:** 2021-06-02

**Authors:** Suguru Kimura, Naoki Miyamoto, Kenneth L. Sutherland, Ryusuke Suzuki, Hiroki Shirato, Masayori Ishikawa

**Affiliations:** ^1^ Department of Medical Physics Graduate school of Medicine Hokkaido University Sapporo Hokkaido Japan; ^2^ Division of Radiological Technology National Hospital Organization Hokkaido Cancer Center Sapporo Hokkaido Japan; ^3^ Faculty of Engineering Hokkaido University Sapporo Hokkaido Japan; ^4^ Global Center for Biomedical Science and Engineering Faculty of Medicine Sapporo Hokkaido Japan; ^5^ Department of Medical Physics Hokkaido University Hospital Sapporo Hokkaido Japan; ^6^ Department of biomedical science and Engineering Faculty of Health science Hokkaido University Sapporo Hokkaido Japan

**Keywords:** image quality, quality assurance, quality assurance for imaging devices, quality control, real‐time tumor tracking

## Abstract

**Purpose:**

The real‐time tumor tracking radiotherapy (RTRT) system requires periodic quality assurance (QA) and quality control. The goal of this study is to propose QA procedures from the viewpoint of imaging devices in the RTRT system.

**Methods:**

Tracking by the RTRT system (equips two sets of colored image intensifiers (colored I.I.s) fluoroscopy units) for the moving gold‐marker (diameter 2.0 mm) in a rotating phantom were performed under various X‐ray conditions. To analyze the relationship between fluoroscopic image quality and precision of gold marker coordinate calculation, the standard deviation of the 3D coordinate (σ3D [mm]) of the gold marker, the mean of the pattern recognition score (PRS) and the standard deviation of the distance between rays (DBR) (σDBR [mm]) were evaluated.

**Results:**

When tracking with speed of 10‐60 mm/s, σDBR increased, though the mean PRS did not change significantly (p>0.05). On the contrary, the mean PRS increased depending on the integral noise equivalent quanta (∫NEQ) that is an indicator of image quality calculated from the modulation transfer function (MTF) as an indicator of spatial resolution and the noise power spectrum (NPS) as an indicator of noise characteristic.

**Conclusion:**

The indicators of NEQ, MTF, and NPS were useful for managing the tracking accuracy of the RTRT system. We propose observing the change of these indicators as additional QA procedures for each imaging device from the commissioning baseline.

## 1 INTRODUCTION

Real‐time tumor tracking radiotherapy (RTRT), proposed in 1999 by Shirato et al., is a high accurate radiotherapy method. With this technique, the motion of a surrogate gold marker inserted into the tumor's vicinity is serially tracked at a rate of 30 frames/s using a pair of fluoroscopic devices. The radiation treatment beam is applied only when the gold marker is located within a preset range.^1^, ^2^ This technique was originally designed as a means of gated irradiation primarily for organs showing respiration‐related motion such as lung and liver, but its clinical use has been contributing to the reduction of planning target volume margins and improvement of patient localization based on the gold marker.^3^

Quality assurance (QA) and quality control are important for radiotherapy. The American Association of Physicists in Medicine (AAPM) Task Group 142 (TG‐142) Report (2009) describes the recommended methods specifically.^4^ The lists of accessories for radiation treatment devices given in that report include radiographic imaging. The RTRT system is one of the “planner kV imaging” devices. In that section of the report, the recommended QA procedures (geometric accuracy, image quality, fluoroscopic dose, etc.) are described clearly. In focusing on the image quality item (spatial resolution, contrast, uniformity, and noise) in the report, the baseline data from the commissioning are recommended as criteria for QA. In addition, the RTRT system also has the aspect of an X‐ray device for use in diagnostic radiology. As a standard metric for this class of device, the International Electrotechnical Commission (IEC) 62220‐1 Standard (2003) is now used extensively. This standard describes the methods of image quality evaluation with quantitative indicators of digital imaging devices for medical use, involving the evaluation of the modulation transfer function (MTF) as a resolution characteristic, the noise power spectrum (NPS) as a noise characteristic, and the detective quantum efficiency (DQE) as a detector performance.^5^

The RTRT system has recently undergone several improvements. SyncTraX (Shimadzu Corporation, Japan), with a colored image intensifier (II), began to be used clinically at Hokkaido University Hospital in July 2014.^6^ SyncTraX was jointly developed by Hokkaido University as a general‐purpose RTRT system and can be linked to a Varian Medical Systems linear accelerator. To date, independent verification of its geometric accuracy and tracking performance has been carried out within the framework of QA of this system, but the association between three‐dimensional (3D) tracking accuracy and image quality has not been analyzed. In addition, this system differs markedly from ordinary imaging devices for medical use in terms of the object’s geometric system and purposes of use.

The present study was performed to propose QA procedures for this system, which covering all of the AAPM TG‐142 planner kV imaging items among the fluoroscopic image quality applicable to radiation treatment devices and assuring satisfactory accuracy of 3D tracking of gold markers with this system. For this purpose, we analyzed the relationship between the fluoroscopic image quality and the accuracy of the gold marker coordinate calculation using characteristic indicators for the RTRT system.

## 2 METHODS

### 2.A Real‐time tumor tracking radiotherapy system specifications

**Fig. 1:**
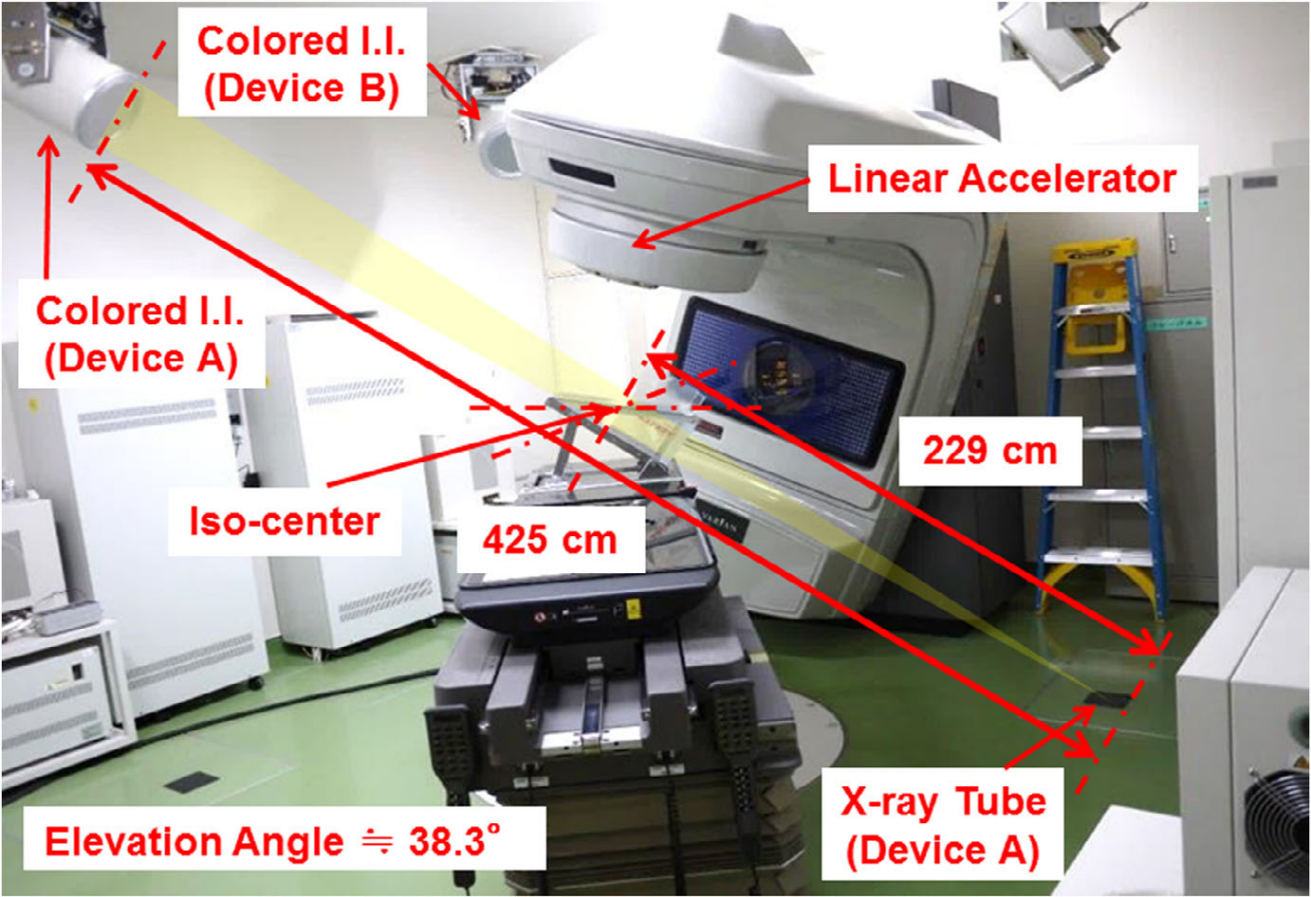
The prototype SyncTraX used in the present study. Device A and Device B are both composed of an X‐ray tube installed under the floor and a colored II installed on the ceiling. The experimental geometry for device A is shown.

The present study used the current prototype SyncTraX. As shown in Fig. 1, the prototype system consists of two pairs of fluoroscopic devices (Device A and B), a pulse controller, and a host personal computer for image capture, pattern matching, and coordinate calculation. Devices A and B are both composed of an X‐ray tube installed under the floor and a colored II installed on the ceiling, with the center of the X‐ray axes intersecting at the isocenter.^2^ The geometric parameters are also displayed in Fig. 1. The X‐ray tube enables free setting of the tube voltage in the range 40–110 kV, the tube current in the range 10–200 mA, and the pulse width in the range 1–4 ms. The colored phosphor unit is made of Y_2_O_2_S:Eu. The light emitted from this unit is quantized into three components (red, green, and blue [RGB]), each consisting of 8 bits (0–255 gradations).^6^ The actual field of view (FOV) of the colored II is 228.6 mm. The gold marker at the isocenter is magnified by the geometric system. Thus, the imaging area is calculated to be 123.18 mm in diameter around the isocenter. This area is defined as the effective field of view (EFOV) as opposed to the actual FOV. Because the charge‐coupled devices (CCD) has a resolution of 1000 × 1000 pixels for the EFOV, the pixel size of the colored II is deemed to be 0.123 mm.

With the RTRT system, the 3D coordinates of the gold marker are calculated at a rate of 30 frames/s from a pair of two‐dimensional (2D) fluoroscopic images. The 2D coordinates of the gold marker on the fluoroscopic images are determined by pattern matching. Pattern matching employs a 2D model image (usually 24 × 24 pixels) of the gold marker registered with the software in advance as a template image. The coordinates with the highest pattern recognition score (*PRS*) based on the normalized cross‐correlation within the search area (usually 64 × 64 pixels) adopted as the 2D coordinates of the gold marker. The normalized cross‐correlation formula *PRS* is given by

(1) $$PRS=100\times {\left(\frac{N\sum_{i=1}^{N}F_{i}G_{i}‐\left(\sum_{i=1}^{N}F_{i}\right)\sum_{i=1}^{N}G_{i}}{\sqrt{\left|N\sum_{i=1}^{N}F_{i}^{2}‐{\left(\sum_{i=1}^{N}F_{i}\right)}^{2}\right|\left|N\sum_{i=1}^{N}G_{i}^{2}‐{\left(\sum_{i=1}^{N}G_{i}\right)}^{2}\right|}}\right)}^{2}.$$

**Fig. 2:**
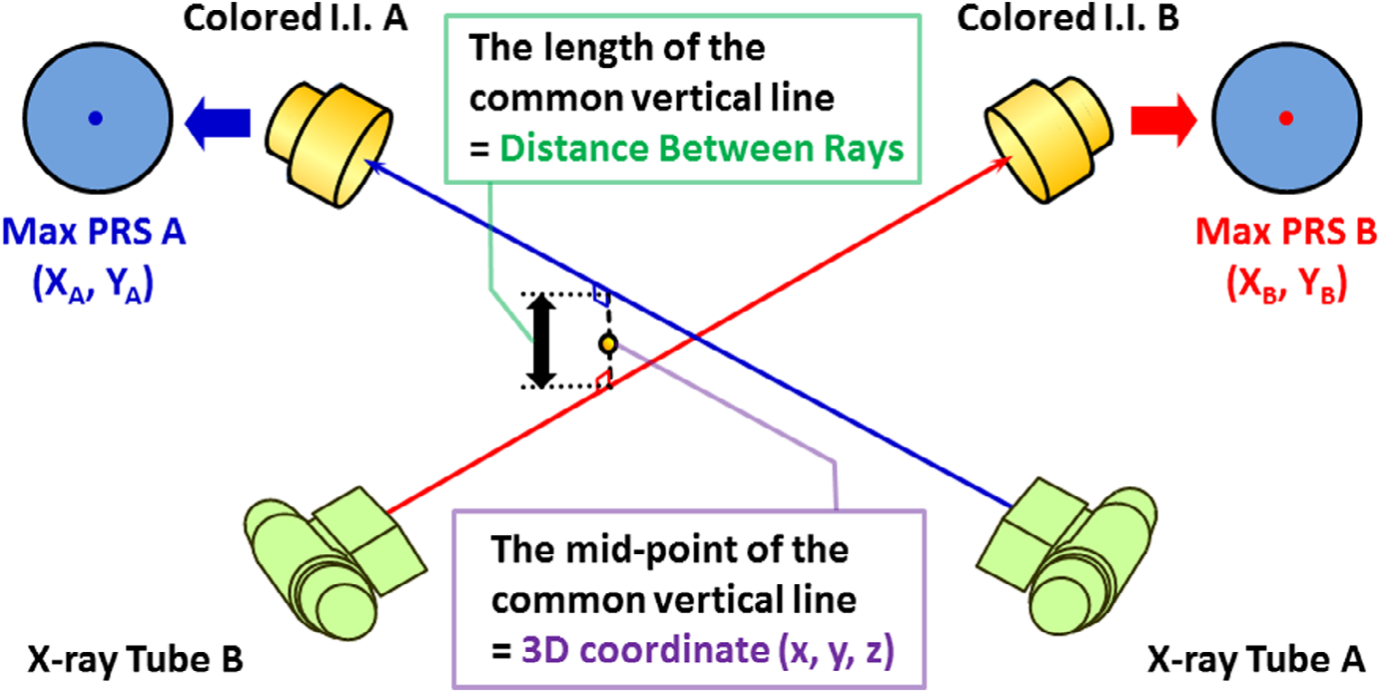
Schematic illustration of the common vertical line connecting the two spatial vectors joining the X‐ray tube focus. The midpoint of the common vertical line is used as the 3D coordinates of the gold marker.

Here, *N* is the total number of pixels, *G_i_* is the pixel values of a template image, and *F_i_* is the pixel values of a fluoroscopic image. The *PRS* ranging from 0 to 100 is calculated from multiplying 100 by the square of the normalized cross‐correlation and is set to 0 when the normalized cross‐correlation is negative.^2^, ^7^ In this process, the size of the tracked gold marker (diameter 1.5–2.0 mm) needs to be matched to the template image. With the colored II having three components (RGB), the *PRS* is calculated separately for each component, and the 2D coordinates for the component having the highest *PRS* are used. As shown in Fig. 2, the midpoint of the common vertical line connecting these vectors is used as the 3D coordinates of the gold marker, and the length of this common vertical line is defined as the distance between rays (*DBR*).

### 2.B Experimental procedures and tools

**Fig. 3:**
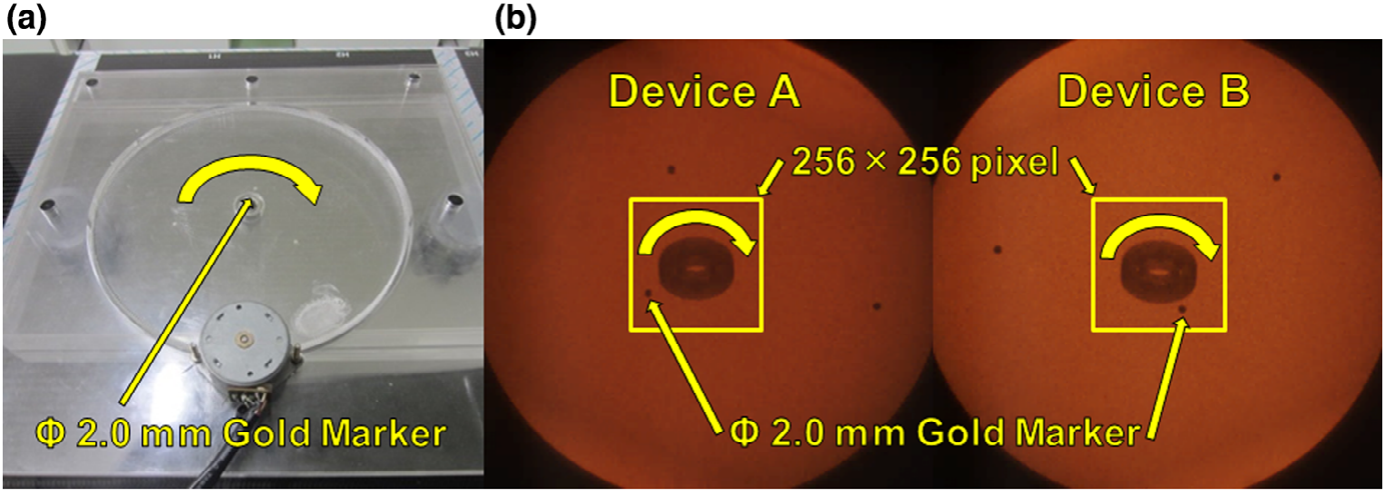
Rotating phantom embedded with three gold fiducial markers. (a) Actual image and (b) fluoroscopic image.

A rotating phantom was used for tracking accuracy verification (Fig. 3). The relationship between *PRS*, *DBR*, and the fluoroscopy settings was analyzed. The rotating phantom was made of PMMA (300 × 300 × 14 mm, about 1.2 g/mm^3^ in density), and 2‐mm*ϕ* gold markers were embedded in the rotating disk at points 16, 40, and 48 mm from the disk center. Total thickness of PMMA was 10 cm using additional PMMA to simulate an actual patient body. The equivalent path length was 16.13 cm of PMMA at the center of the X‐ray axis.

The relationship between the 2D fluoroscopic image quality and PRS was then analyzed. A phantom made of PMMA (30 × 20 × 0.2 cm^3^) was placed at the isocenter as a support to the line pair chart (spatial frequency: 0.5–6.0 lp/mm, thickness: 0.1 mm Pb, Kyokko Type 8) for resolution evaluation and the 2.0‐mm*ϕ* gold markers for PRS evaluation. Some phantoms made of PMMA (30 × 20 × 5 cm^3^) were placed sandwiching the isocenter to simulate actual patient body. The PMMA phantom was aligned vertically to the X‐ray axis. ImageJ (National Institutes of Health, USA) was used for image processing including pixel value extraction, angle measurement on fluoroscopic images, division of fluoroscopic images into RGB components, stacking of multiple images, preparation of averaged images, preparation of a differential image from a pair of images, and the fast Fourier transform (FFT) of cropped images.

A 150‐cm^3^ ionization chamber (Type 96020C, IBA Dosimetry) with an electrometer (TRIAD TnT Dosimeter, Fluke Biomedical, USA) was used for the dose rate measurement which is required for the color II's input/output characteristics.

### 2.C 3D tracking accuracy and *PRS*/*DBR*

Safety of synchronized irradiation is assured by control of the linear accelerator through indicator‐based interlocking. *PRS* and *DBR* are used as indicators for this purpose and are recorded in the tracking log together with the gold marker's 3D coordinates. Because the gold marker position is decided as the midpoint of the common vertical line between the two tracking vectors, the gold marker's 3D tracking discrepancy accords with a distance half that of *DBR*. For example, if the *DBR* is within 2.0 mm during tracking, the deviation in the 3D coordinates is within 1.0 mm (equivalent to half of the gold marker size). The present study was designed to determine mean *PRS* when the 3D tracking accuracy was set within the lower limit of 1.0 mm and the objective limit of 0.5 mm, that is, when the threshold was set at one‐sided accuracy 0.5 mm and 0.25 mm. Figure 3b shows the fluoroscopic images of the rotating phantom of Devices A and B. The gold marker's 2D coordinates are continuously calculated from the highest *PRS* within the search area. During gold marker tracking, the *PRS* of each component (RGB) of Devices A and B and the calculated 2D coordinates are recorded in the tracking log. On the basis of calculation of 2D coordinates with both Devices A and B, 3D coordinates of the gold marker and *DBR* are calculated. First, we evaluated the relationship of the 3D tracking accuracy to *DBR* and *PRS* needed for one side of Devices A and B. The setting for fluoroscopy of one device was fixed at 80 kV, 200 mA, 4 ms as a reference fluoroscopic condition, while the other device was fixed at pulse width 4 ms under the fluoroscopy setting of tube voltage 70, 80, and 90 kV and tube current 10–200 mA. In this way, the still gold marker was tracked with the use of a phantom with total PMMA thickness 10 cm (simulation of a standard physique). From the tracking log covering the 300 frames under the tracking possible setting of fluoroscopy, we calculated the *PRS* and *DBR* of both devices as well as changes in 3D coordinates (σ_3D_) using the following formula:

(2) $$\sigma_{3D}=\sqrt{\sigma_{x}^{2}+\sigma_{y}^{2}+\sigma_{z}^{2}}.$$

Here, σ_x_, σ_y_, and σ_z_ denote the standard deviation (SD) of the 3D coordinates (x, y, z) for 300 frames. When the object is still, zero is ideal, but in practice, 3D statistical variations are present. In this study, keeping the 3D tracking accuracy within 0.5 mm at a probability of 99% was set as the goal, with the lower limit set at 3σ_3D_ < 0.5 mm and the objective limit set at 3σ_3D_ < 0.25 mm. Based on such constraints, the relationship of 3σ_3D_ to the SD of *DBR* (σ*_DBR_*) and the *PRS* on one side was evaluated for Devices A and B. However, for the colored II with which 2D coordinates were calculated based on maximum *PRS* of RGB, the mean and SD of maximum *PRS* for RGB were evaluated.

Furthermore, under the same fluoroscopy setting, the gold marker was tracked for 10 s or more at varying rotation rates of the phantom in the range of 0–60 mm/s (at intervals of 10 mm/s). The data from a 10‐s period (300 frames) were extracted in this experiment. The log was used to examine if the mean *PRS* would differ between the still state and the moving state under Device A and B setting of tube voltage 80 kV, tube current 200 mA, and pulse width 4 ms. We performed a multiple comparison test for nonparametric data (Shirley–Williams method) between the still state (0 mm/s) and the moving state (10–60 mm/s). In this experiment, the phantom was set so that the range of gold marker motions would remain in the central 256 × 256 pixel region. This setting was intended to check that the mean *PRS* was comparable. In this case, the gold marker with motion did not allow evaluation of σ_3D_, and so, the correlation of the mean *PRS* and the marker speed to the σ*_DBR_* under the identical fluoroscopy settings were evaluated.

### 2.D Fluoroscopic image quality and *PRS*

We analyzed the relationship between the 2D fluoroscopic image quality and *PRS* so that we could evaluate one part of this system. Whereas the 2D coordinates from two devices are necessary for calculation of the *DBR*, the *PRS* is provided in each RGB components from each device.

MTF is used as an indicator of resolution characteristic of digital imaging devices for medical use. The IEC 62220‐1 recommends the edge method for the evaluation of MTF^5^; however, we applied a method such that the ROI is set on the image taken with approximately 45° inclination of the line pair chart and the MTF is evaluated on the basis of the mean and SD of its pixel value.^8^, ^9^, ^10^, ^11^ Since this method enables evaluation while placing the line pair chart at the isocenter with the use of the linear accelerator couch, the procedure is optimal for the geometric system. The formula used for calculation of MTF is given below.^8^

(3) $$\text{MTF}\left(f\right)=\left(\frac{\pi }{2}\right)\frac{\sqrt{2\sigma_{f}^{2}‐\left(\sigma_{a}^{2}+\sigma_{t}^{2}\right)}}{\left|m_{a}‐m_{t}\right|}\;\left(f\gg \frac{f_{c}}{3}\right)$$

Here, σ_f_ denotes the SD of the ROI's pixel value in the spatial frequency (f) line pair; σ_a_ and σ_t_ indicate the SD of the ROI's pixel value for the uniform 0.1 mm Pb and the homogeneous background, respectively; and m_a_ and m_t_ are the mean of the ROI's pixel value for the homogeneous 0.1 mm Pb and the uniform background, respectively. The line pair chart was attached to the 2‐mm PMMA layer and placed at the isocenter at an angle of 45 ± 1°. Because the pixel width of the colored II was 0.123 mm, the theoretical MTF reaches 0 in the vicinity of the Nyquist frequency of about 4.0 lp/mm. If the cutoff frequency is denoted as f_c_, the spatial frequency which can be evaluated with formula (3) is about 1.35 lp/mm or less. So the spatial frequency which can be evaluated is 0.5, 0.75, 1.0, and 1.25 lp/mm.

**Fig. 4:**
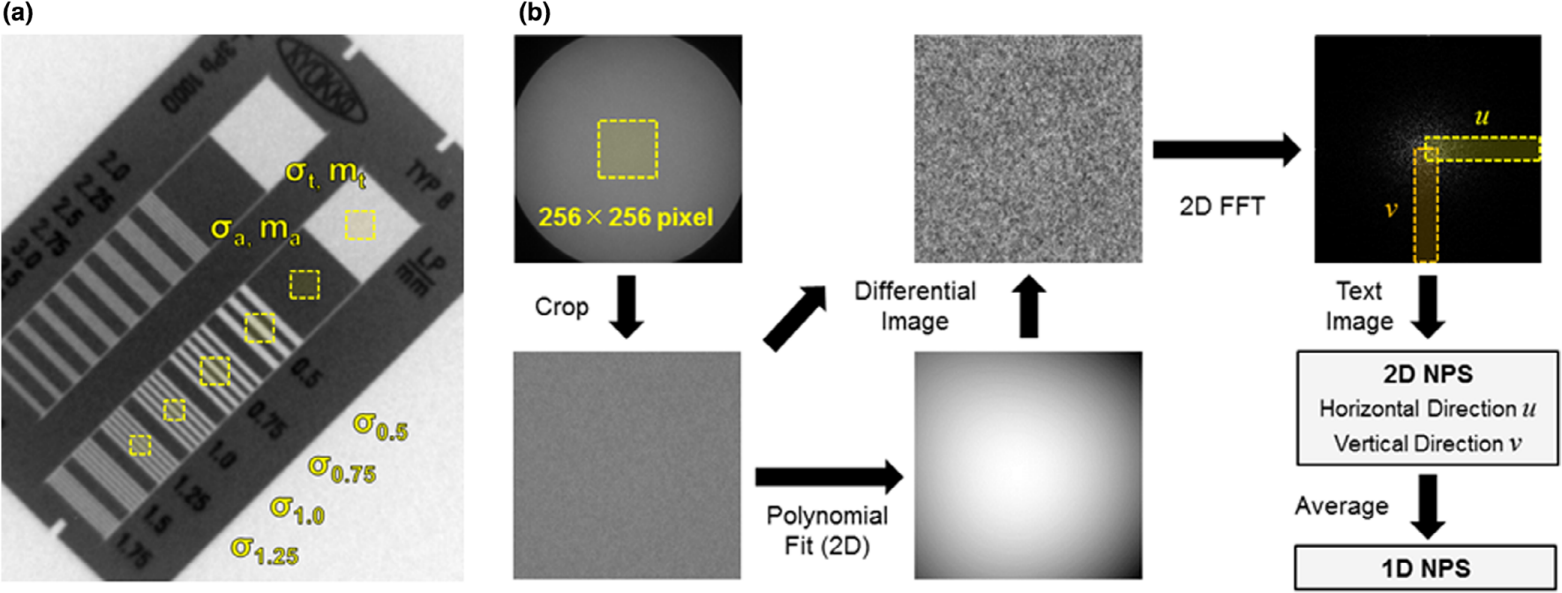
Schematic illustration of image processing with ImageJ. (a) Image of a line pair chart at an angle of 45 ± 1°. 30‐ or 20‐pixel square ROIs were set on the fluoroscopic images. (b) Flowchart of image processing for 1D NPS evaluation.

We first evaluated input/output characteristics of II, the air kerma rate (μGy/frame) was measured at the isocenter with tube voltage of 80 kV, which is the recommended tube voltage for the line‐pair evaluation. With multiple combinations of tube current (10, 25, 50, 80, 100, and 200 mA) and pulse width (1, 2, 3, and 4 ms), air kerma (μGy) for 30 s was measured five times, and the air kerma rate was calculated. The mean pixel value was obtained for the central 256 × 256 pixels of five fluoroscopic images taken under the fluoroscopy setting identical to that for air kerma rate measurement. On the basis of the characteristic curve, the fluoroscopy setting in which each of the RGB pixel values was closest to 128 (the center of the 8‐bit range, 0–255) was selected for the highest precision evaluation of MTF. Because the precision of measuring MTF can be improved by the use of averaged images containing little noise, we prepared 10 averaged images from 100 fluoroscopic images of the line pair chart obtained with Devices A and B. Each of these averaged images was evaluated with the mean ± SD of MTF.^11^ Figure 4a shows the ROI set on the fluoroscopic images. As described above, the ROI was set in the line pair with the spatial frequency 0.5, 0.75, 1.0, and 1.25 lp/mm. The size of the ROI was matched to the line pair width, adopting a 30‐pixel square for σ_a_, σ_t_, σ_0.5_, and σ_0.75_ and a 20‐pixel square for σ_1.0_ andσ_1.25_.

NPS is used as an indicator of the noise characteristic of digital imaging devices for medical use.^12^, ^13^ In the IEC 62220‐1, 2D FFT of the 256 × 256 pixel ROI on a uniform image is recommended for evaluation of NPS. The noise components of a uniform image have two dimensions (horizontal and vertical) and can be visualized in the spectrum of spatial frequency intervals determined by the pixel size. The formula for calculation of NPS defined in IEC 62220‐1 is shown below.^5^

(4) $$NPS\left(u,v\right)=\frac{\Delta x∙\Delta y}{M∙256∙256}\sum_{m=1}^{M}{\left|\sum_{i=1}^{M}\sum_{j=1}^{M}\left\{I\left(x_{i},y_{i}\right)‐S\left(x_{i},y_{i}\right)\exp\left(‐2\pi i\left(ux_{i}+v\right)\right)\right\}\right|}^{2}\;$$

Here, ∆x and ∆y indicate the pixel size of vertical and horizontal axes, and M denotes the number of ROIs evaluated. I(x_i_, y_j_) is the pixel value of the 256 × 256 pixel ROI to be evaluated. S(x_i_, y_j_) is the curved surface approximating the quadratic polynomial equation. The subtraction shown in this formula allows correction of the trend. Each possible combination of tube voltage (80 kV), tube current (10, 25, 50, 80, 100, and 200 mA) and pulse width (1, 2, 3, and 4 ms) was adopted as the fluoroscopic setting for acquisition of images used for the evaluation of NPS. For simulating a standard patient, a PMMA thickness of 10 and 20 cm with the tube voltage of 80 kV was applied. For simulating a larger patient, a PMMA thickness of 30 cm with the tube voltage of 110 kV was applied. From the 100 fluoroscopic images, NPS was evaluated using formula (4), and 100 NPS spectra were averaged to improve the precision of measurement. Because the pixel size of the colored II is 0.123 mm, NPS is expected to be determined for the spectral range from the spatial frequency interval 0.032 to 4.027 lp/mm. Taking into account consistency with MTF evaluation, we evaluated one‐dimensional (1D) NPS by averaging the 2D NPS. A flowchart of the procedure for evaluation is shown in Fig. 4b.

According to the IEC 62220‐1, the next step is evaluation of DQE as a detector performance. However, the actual purpose was the evaluation of the fluoroscopic image quality yielded with a combination of specific fluoroscopy settings and phantom thickness. In this connection, there are reports on the evaluation of noise equivalent quanta (NEQ) as an indicator of image quality before evaluation of DQE based on MTF and NPS.^14^, ^15^ NEQ with following formula (5) is an image quality indicator encompassing elements of resolution, noise, and contrast and is expressed in spatial frequency spectrum as is the case with MTF and NPS.

(5) $$NEQ\left(f\right)=\frac{S^{2}∙{\mathrm{MTF}}^{2}\left(f\right)}{NPS(f)}=\frac{{\mathrm{MTF}}^{2}\left(f\right)}{NNPS(f)}$$

In this formula, *f* denotes spatial frequency, and S^2^ indicates the mean pixel value of the ROI in the fluoroscopic image after evaluation of signals (i.e., NPS). NNPS means the normalized NPS calculated by dividing the NPS by S^2^ and is used for comparison of noise characteristics between images with different mean pixel values. Because the spatial frequency evaluated for MTF was 0.5, 0.75, 1.0, and 1.25, we determined the NPS at the identical spatial frequency using simple linear interpolation, followed by evaluation of NEQ for these four spatial frequencies. Furthermore, to summarize them into a single indicator of fluoroscopic image quality, evaluation was made on the integral noise equivalent quanta (∫NEQ) corresponding to the total of the three rectangular areas surrounded by the four spatial frequencies between 0.5 and 1.25 lp/mm.^11^ Using the data on NEQ, we evaluated the ∫NEQ on each combination of Devices A and B, RGB, fluoroscopy setting, and PMMA thickness.

Furthermore, the gold marker at the isocenter was tracked under the fluoroscopy setting identical to that for NPS evaluation. A hole was created at the center of the 2‐mm‐thick PMMA layer used for NPS measurement, and a 2.0‐mm‐diameter gold marker was installed therein. This experiment was the same as the procedure for obtaining fluoroscopic images in that the 2‐mm‐thick PMMA layer at the isocenter was sandwiched with a 5‐cm‐thick PMMA layer. We prepared an identical experimental system for both Devices A and B and collected the tracking logs for evaluation of mean *PRS* per 100 frames. Finally, we evaluated the relationship between ∫NEQ and mean *PRS*.

## 3 RESULTS

### 3.A Relationship between 3D tracking accuracy and *PRS*/*DBR*

**Fig. 5:**
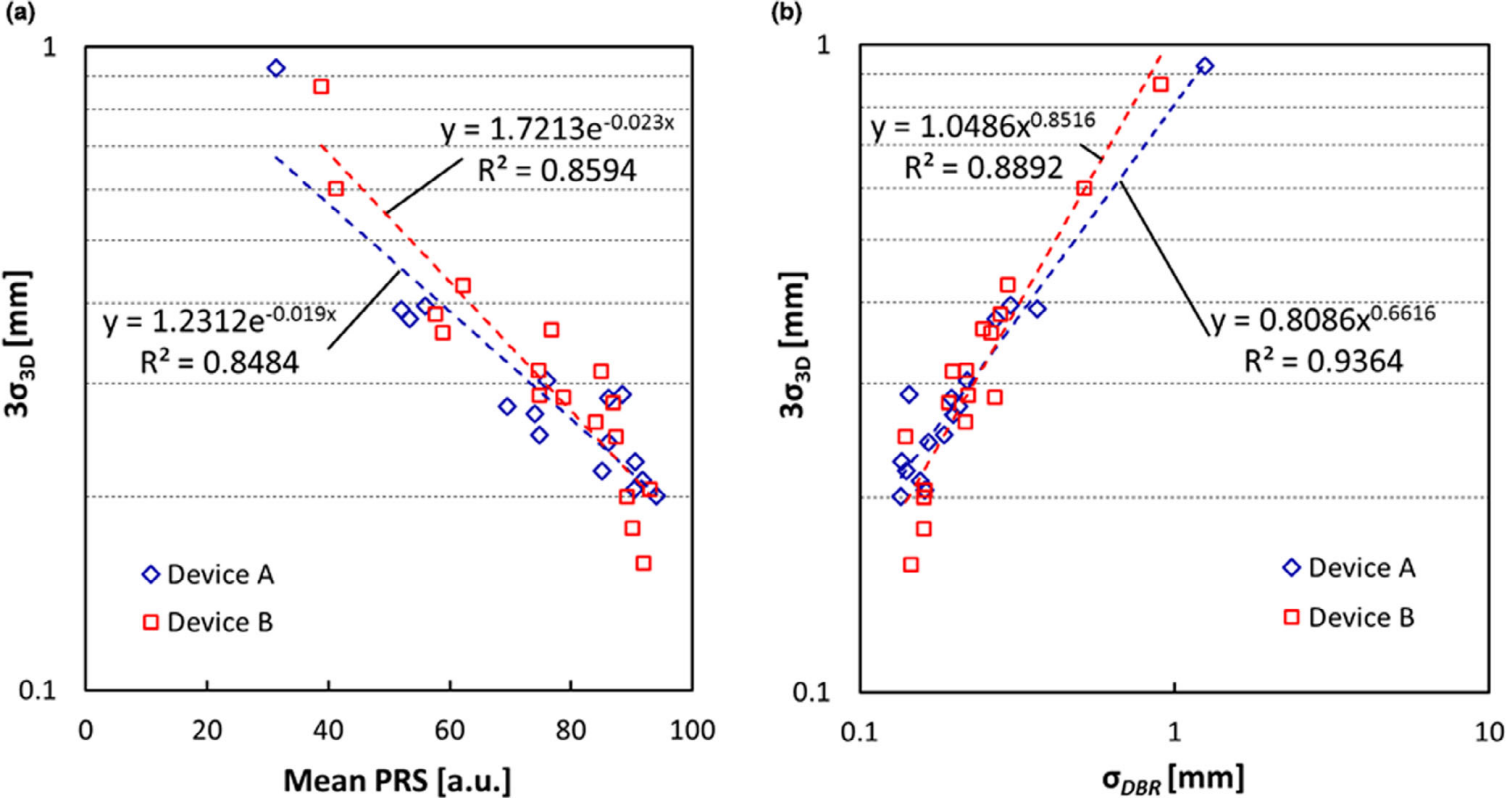
The relationship between the SD of 3D coordinate of a gold marker (3σ_3D_) and unilateral (a) the mean *PRS* and (b) the σ*_DBR_* with various X‐ray conditions and speed of 0 mm/s.

First, we confirmed that the 3D tracking accuracy was within 3σ_3D_ < 0.25 mm (σ_3D_ < 0.076 mm) when we assumed 80 kV, 200 mA, and 4 ms equivalence as a reference fluoroscopic condition in both devices. Figure 5a,b shows the relationship between the 3D tracking accuracy for the still gold marker (3σ_3D_) and unilateral mean *PRS* and σ*_DBR_*, respectively. A negative correlation between *PRS* and 3σ_3D_ is seen in Fig. 5a, and mean *PRS* > 54.34 (Device A) and mean *PRS* > 57.29 (Device B) were needed to achieve the lower limit of tracking accuracy 3σ_3D_ < 0.5 mm when the object was still. To achieve the objective limit 3σ_3D_ < 0.25 mm, mean *PRS* > 80.31 (Device A) and mean *PRS* > 82.27 (Device B) were needed. In Fig. 5b, positive correlation is noted between σ*_DBR_* and 3σ_3D_, and σ*_DBR_* < 0.45 mm (Device A) and σ*_DBR_* < 0.39 mm (Device B) were needed to achieve the lower threshold of tracking accuracy 3σ_3D_ < 0.5 mm when the object was still. To achieve the objective limit 3σ_3D_ < 0.25 mm, σ*_DBR_* < 0.15 mm (Device A) and σ*_DBR_* < 0.17 mm (Device B) were needed.

Table 1 shows the mean *PRS* of the gold marker during the still state and during motion at a speed of 10–60 mm/s (evaluated with the phantom) with Device A and B setting of tube voltage 80 kV, tube current 200 mA, and pulse width 4 ms. With a multiple comparison test, there was no significant difference between the still state and the moving state by the speed in mean *PRS* (*p* > 0.05).

**Table 1:** Mean *PRS* for 2‐mm fiducial gold marker at still state and the moving state.

Marker speed (mm/sec)	Device A				Device B			
	Mean	SD	Max	Min	Mean	SD	Max	Min
0 (Still)	90.34	1.27	93.30	85.00	93.11	1.05	95.80	89.40
10	90.40	1.82	94.50	85.00	92.05	1.52	95.80	88.00
20	90.29	1.97	94.80	82.20	92.01	1.63	96.60	87.10
30	90.14	1.87	94.20	84.20	92.00	1.76	96.10	85.40
40	90.33	1.96	94.50	82.30	92.04	1.60	95.90	87.80
50	90.24	1.84	94.80	85.10	91.82	1.70	95.40	86.30
60	89.90	1.94	94.70	84.30	91.87	1.78	96.00	85.50

In the mean *PRS*, statistical significance difference between the still state and the moving state was not observed in multiple comparison test for nonparametric data (Shirley–Williams method, *P* > 0.05).

**Fig. 6:**
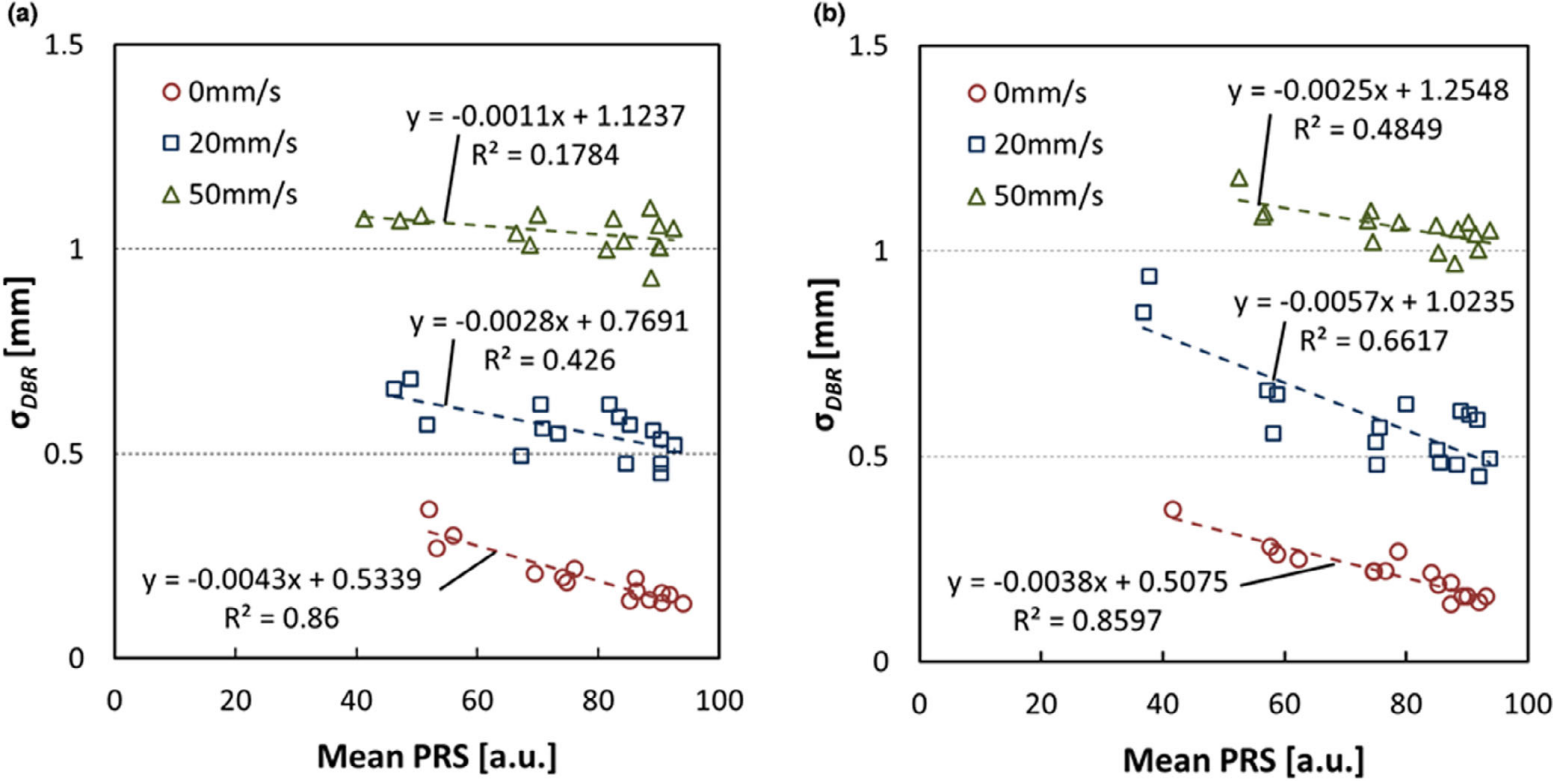
The relationship between the mean *PRS* and the σ*_DBR_* when the setting for fluoroscopy of one device was fixed at 80 kV, 200 mA, and 4 ms of (a) Device A, while the other device (b) Device B was fixed at pulse width 4 ms.

Figure 6 shows the relationship between the mean *PRS* and the σ*_DBR_* when the fluoroscopy setting of one device was fixed at 80 kV, 200 mA, and 4 ms of (a) Device A, while the other device (b) Device B was fixed at pulse width 4 ms under the fluoroscopy setting of tube voltage 70, 80, and 90 kV and tube current 10–200 mA. When we plot the marker speed of 0, 20, and 50 mm/s, there was negative correlation of the mean *PRS* to σ*_DBR_*, but an increase in σ*_DBR_* was shown to occur under the influence of the marker speed rather than the mean *PRS*.

**Fig. 7:**
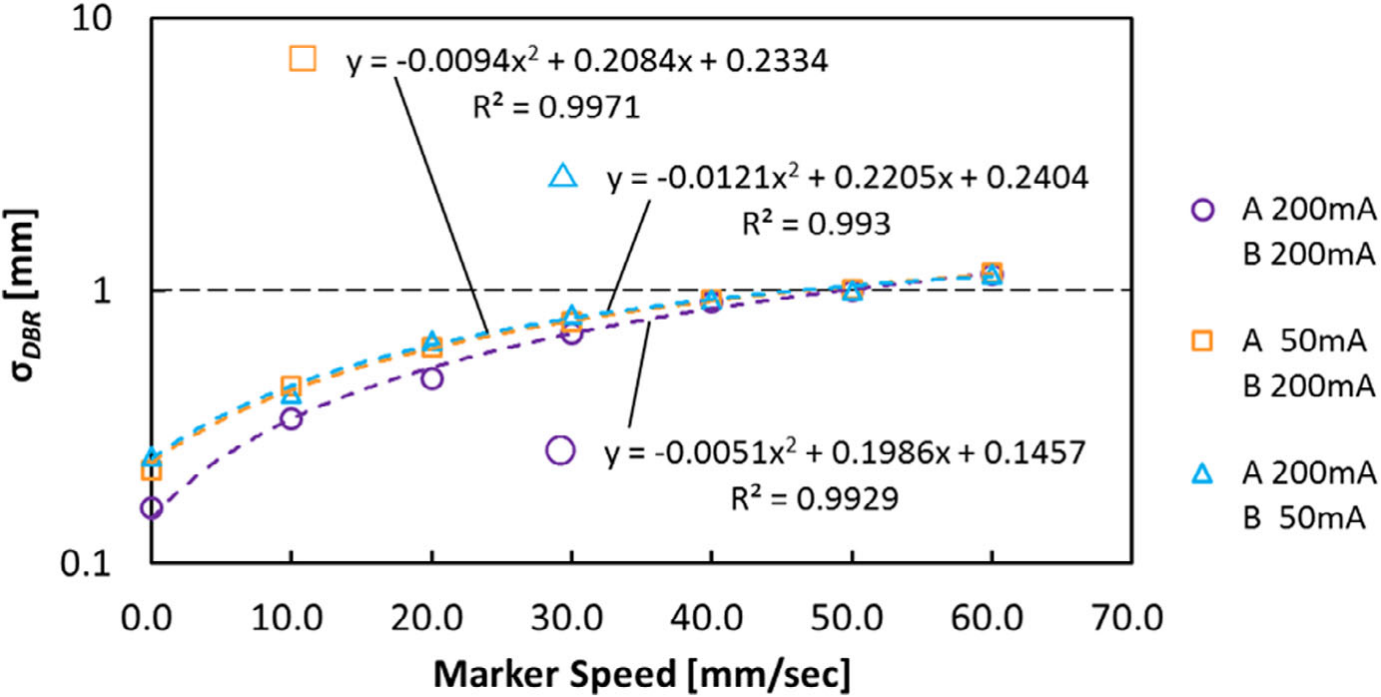
The relationship between the gold marker speed and σ*_DBR_* with Device A and B setting of tube voltage 80 kV, tube current 200 mA (50 mA on one side), and pulse width 4 ms.

Figure 7 shows the relationship between the gold marker speed and σ*_DBR_* with Device A and B setting of tube voltage 80 kV, tube current 200 mA, (and 50 mA on one side) and pulse width 4 ms. There was positive correlation between the gold marker speed and σ*_DBR_* regardless of X‐ray conditions. Similarly, reduction of the fluoroscopic imaging condition increasing the σ*_DBR_* regardless in the same marker speed.

### 3.B Relationship between fluoroscopic image quality and *PRS*

**Fig. 8:**
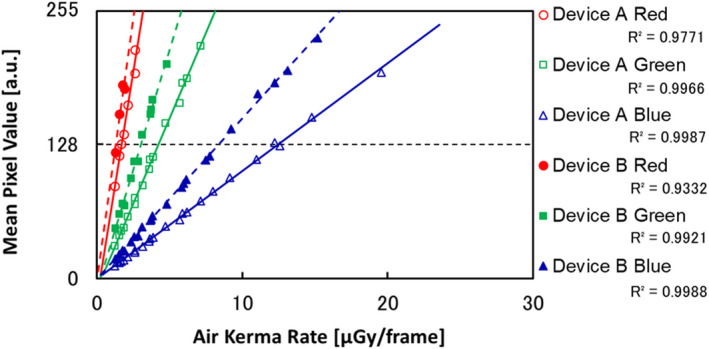
The input/output characteristics of RGB components for each device.

Figure 8 shows the input/output characteristics determined from the air kerma rate measurement and fluoroscopic image pixel values. This characteristic curve indicates that all components (RGB) of Devices A and B form a linear system and that evaluation of MTF and NPS is possible by direct use of pixel values. There were interindividual differences in the input/output characteristics of Devices A and B, but it was possible to get the mean pixel value close to 128 (center of the 8‐bit range, 0‐255), that is, the range from 116.25 ± 0.98 to 137.44 ± 1.21, with each device.

Table 2 shows the MTF and the mean pixel value of fluoroscopic images used for evaluation. In analysis of the SD of MTF determined from 10 averaged images, the error of measurement was small (always less than 2%) even at the highest spatial frequency of 1.25 lp/mm. MTF differed between RGB components of Devices A and B, and each value of MTF was adopted for NEQ evaluation.

**Table 2:** Evaluated MTF and mean pixel value for each device.

Spatial frequency (lp/mm)		0.5	0.75	1.0	1.25	Pixel value
		Mean ± SD	Mean ± SD	Mean ± SD	Mean ± SD	Mean ± SD
Device A	Red	0.734 ± 0.007	0.543 ± 0.005	0.331 ± 0.004	0.167 ± 0.002	128.39 ± 0.44
	Green	0.753 ± 0.003	0.573 ± 0.003	0.387 ± 0.003	0.222 ± 0.003	116.25 ± 0.98
	Blue	0.738 ± 0.003	0.558 ± 0.002	0.357 ± 0.003	0.197 ± 0.002	126.77 ± 0.62
Device B	Red	0.748 ± 0.005	0.571 ± 0.003	0.370 ± 0.005	0.219 ± 0.004	120.12 ± 1.03
	Green	0.746 ± 0.003	0.564 ± 0.003	0.372 ± 0.002	0.233 ± 0.003	137.44 ± 1.21
	Blue	0.744 ± 0.002	0.554 ± 0.003	0.347 ± 0.003	0.222 ± 0.003	116.48 ± 2.65

MTF and mean pixel value from images of the fluoroscopy setting in which each of the RGB pixel values were closest to 128 for the highest precision evaluation of MTF.

When NEQ is evaluated, NPS is often divided in advance by S^2^ of formula (5) to yield NNPS. Table 3 shows the maximum and minimum of NNPS evaluated for each RGB value of Devices A and B yielded from the combination of fluoroscopy setting and PMMA thickness at the spatial frequency 0.5, 0.75, 1.0, and 1.25.

**Table 3:** Maximum and minimum evaluated NNPS of RGB components for each devices.

Spatial frequency (lp/mm)		0.5		0.75		1.0		1.25	
		Max	Min	Max	Min	Max	Min	Max	Min
Device A	Red	1.63 × 10^−3^	1.42 × 10^−4^	1.21 × 10^−3^	9.65 × 10^−4^	8.57 × 10^−4^	5.68 × 10^−5^	5.79 × 10^−4^	3.12 × 10^−5^
	Green	6.12 × 10^−4^	9.65 × 10^−5^	5.22 × 10^−4^	6.20 × 10^−5^	3.94 × 10^−4^	4.46 × 10^−5^	3.26 × 10^−4^	3.12 × 10^−5^
	Blue	8.83 × 10^−4^	8.56 × 10^−5^	8.08 × 10^−4^	6.20 × 10^−5^	6.78 × 10^−4^	4.46 × 10^−5^	5.60 × 10^−4^	3.22 × 10^−5^
Device B	Red	1.67 × 10^−3^	1.46 × 10^−4^	1.30 × 10^−3^	9.72 × 10^−5^	9.59 × 10^−4^	6.47 × 10^−5^	6.59 × 10^−4^	4.19 × 10^−5^
	Green	6.82 × 10^−4^	7.29 × 10^−5^	5.87 × 10^−4^	5.51 × 10^−5^	4.67 × 10^−4^	3.95 × 10^−5^	3.71 × 10^−4^	2.89 × 10^−5^
	Blue	1.12 × 10^−3^	1.10 × 10^−4^	1.00 × 10^−3^	9.03 × 10^−5^	8.16 × 10^−4^	6.74 × 10^−5^	7.28 × 10^−4^	4.73 × 10^−5^

Based on thus evaluated MTF and NNPS, we evaluated the image quality indicator NEQ for combinations of fluoroscopy setting and PMMA thickness at the spatial frequency 0.5, 0.75, 1.0, and 1.25. Maximum and minimum ∫NEQ and the mean *PRS* determined under different settings of the same experimental system are shown in Table 4.

**Table 4:** Maximum and minimum evaluated ∫NEQ and mean *PRS* for each devices.

Index		∫NEQ (a.u.)		Mean *PRS* (a.u.)	
		Maximum	Minimum	Maximum	Minimum
Device A	Red	1830.27	179.57	96.47	65.85
	Green	3183.99	390.54	94.81	64.07
	Blue	2916.08	229.04	92.42	64.60
Device B	Red	1726.77	131.07	96.67	72.95
	Green	3625.28	340.48	96.07	65.30
	Blue	1994.83	187.65	96.00	64.80

**Fig. 9:**
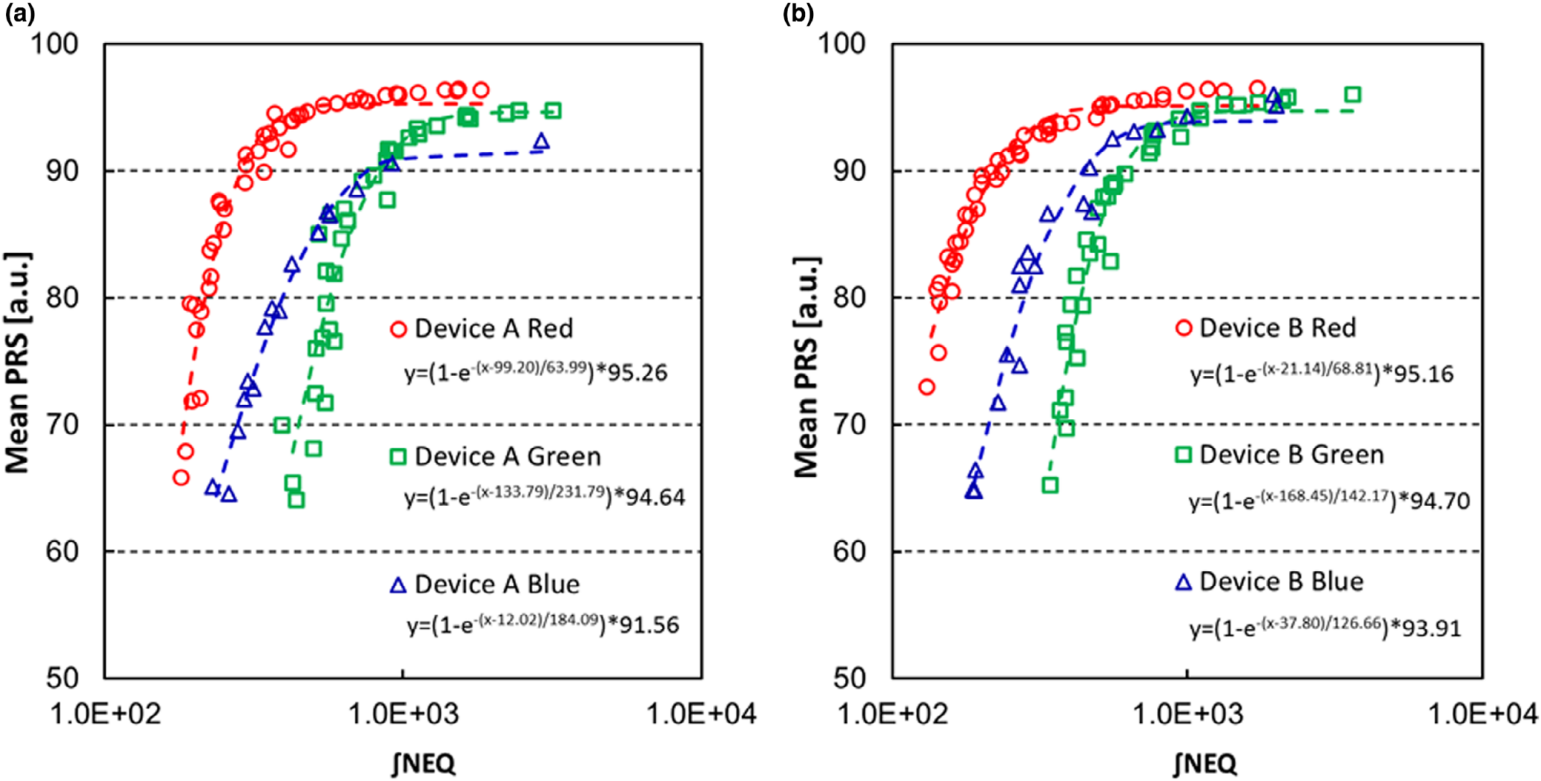
The relationship between the mean *PRS* and ∫NEQ from images of RGB components for each device.

Figure 9 plots the relationship between ∫NEQ and mean *PRS*. This figure indicates that for each RGB value with Devices A and B, mean *PRS* improves as ∫NEQ gets larger. ∫NEQ was thus shown to correlate closely with *PRS*. When tube voltage was set at 80 or 110 kV and the PMMA thickness at 10, 20, or 30 cm, there was no dependency on tube voltage or differences in the object. However, the relationship between image quality and mean *PRS* varied depending on the device and the RGB components. This finding seems to involve interindividual variances in input/output characteristics and differences in spatial frequency characteristics. In other words, since the RTRT system using a colored II has six detectors with different spatial frequency characteristics, verification is needed for each of these detectors.

In this study, if we adopt the lower limit determined in results 3.A, the ∫NEQ for RGB components needed to exceed the lower limit (3σ_3D_ < 0.5 mm) was 136.25, 337.56, and 116.60 with Device A and 52.48, 232.83, and 128.97 with Device B. As well as the objective limit, the ∫NEQ for RGB needed to exceed the objective limit (3σ_3D_ < 0.25 mm) were 223.21, 585.50, and 429.57 with Device A and 162.39, 491.51, and 337.55 with Device B.

## 4 DISCUSSION

When compared with the previous IEC 61267 (1994), the IEC 62220‐1 has shifted to image evaluation methods using objective indicators instead of subjective indicators.^5^, ^16^ In the present study, we evaluated the image quality on the basis of methods in the IEC 62220‐1, though we adopted another more optimal method in several points. The input/output characteristic of this system is proportional with X‐ray exposure, and direct evaluation based on fluoroscopic image pixel value was possible for MTF and NPS evaluation. In evaluation of MTF, the methods adopted in the present study have already been evaluated in published studies on QA of electric portal imaging devices, demonstrating excellent simplicity and efficiency because direct evaluation from images is possible.^11^ However, the NPS defined in IEC 62220‐1 contains the term for correction; we adopted it directly for this study, taking into account also that the quadratic polynomial equation was most effective. 1D NPS was evaluated with averaging of 2D NPS recommended in IEC 62220‐1.^5^ NEQ can be calculated from MTF and NPS of the identical spatial frequency as an image quality indicator. Therefore, we could correlate the tracking accuracy with fluoroscopic images from the RTRT system. The NEQ can be applied to other devices using digital planner kV imaging, but it is necessary to establish QA procedures for each device with characteristics and limitations.

In the results of Fig. 8, there were interindividual differences in the input/output characteristics of Devices A and B. And in the results of Fig. 9, the *PRS* remained small with higher NEQ in Device A blue, and the NEQ needed to exceed the limit was about 1.5–3 times in Device A. These characteristics occurred due to the interindividual variances in II including the colored phosphor unit, the CCD, and the optical lens. The prototype system in the present study was not for a commercial system, lacking detailed adjustments. However, we have confirmed the repeatability in another study using the prototype system. In other words, we can use the indicator also for adjustment of the interindividual difference of fluoroscopic devices.

The mean *PRS* has a relationship with the 3D tracking accuracy under the static condition. However, in the case of images with the mean *PRS* values of 60 or less, the 2D FFT could not be performed due to insufficient pixel values. As a result, NNPS and ∫NEQ could not be calculated, and the relationship between the image quality and the mean *PRS* was investigated in the mean *PRS* of more than 60. Since the real‐time tumor tracking system finally performs the 3D coordinate calculation using the digital value of the fluoroscopic image, it is reasonable that a strong correlation was found between the ∫NEQ and the 3D tracking accuracy. In other words, evaluating the device with an objective indicator is very suitable for this system. If a high NEQ fluoroscopic image can be output at a relatively low‐dose rate, it may be the simplest approach to improving the performance of the device. This method can also be used in the development of detectors with spatial frequency characteristics that increase the 3D tracking accuracy.

According to the results of Fig. 5 (the relationship between 3σ_3D_, mean *PRS*, and σ*_DBR_*), Table 1 (the relationship between the mean *PRS* and the gold marker speed), Fig. 6 (the relationship between mean *PRS* and σ*_DBR_*), and Fig. 7 (the relationship between σ*_DBR_*, the gold marker speed, and the fluoroscopic imaging condition), we found the following facts: (1) The 3D tracking accuracy depended on not only *PRS* but also *DBR*; (2) the mean *PRS* was not changed by the marker speed; (3) the σ*_DBR_* was changed by the marker speed and fluoroscopic condition. Those results indicated that the mean *PRS* was a necessary condition for 3D tracking accuracy of the gold marker regardless with or without motion. In addition, the mean *PRS* was able to determine by every device unlike the σ*_DBR_*; it was possible to relate with the 2D image quality.

To date, relations of the coordinate calculation precision in the 2D fluoroscopic image have already become clear for the mean *PRS* in the past study, but not for the 3D tracking accuracy.^17^ It is difficult to correlate 3D tracking accuracy to 2D fluoroscopic image quality directly; therefore, we tried to assess the 3D tracking accuracy from the mean *PRS* and the relationship between the mean *PRS* and ∫NEQ. It has been shown that the mean *PRS* improves as the ∫NEQ becomes higher, and as a result, the 3D tracking accuracy improves. On the other hand, since the mean *PRS* is not significantly affected by the moving speed of the marker, we also investigated the relationship between σ*_DBR_* and 3D tracking accuracy. Since σ*_DBR_* is affected by the marker moving speed, it is desirable to use two indices, ∫NEQ as the image quality and σ*_DBR_* as the maximum traceable speed, when evaluating the 3D tracking accuracy.

Table 5 gives a cross‐reference between part of the QA items for image quality in AAPM TG‐142 planner kV imaging and the image quality indicators evaluated with the fluoroscopic device in the present study. As a result, all items related to indicators of images were filled with objective indicators, allowing checking of changes from the baseline data collected at the time of commissioning. Furthermore, if multiple indicators are combined, some extended indicators may be created, possibly leading to more reliable QA. Of the items listed in AAPM TG‐142 planner kV imaging, only imaging dose is designed for annual check. However, it seems desirable to check this item at the same timing as the check of other items which need to be made monthly so that confirmation based on input/output characteristics may be enabled as to which of the deterioration in X‐ray tube or detector over time is causing changes under the nominal fluoroscopy setting.

**Table 5:** Cross‐reference between QA procedures and image quality indicators.

Procedure (frequency)	Indicator	Extended indicator
Imaging dose (annual)	Air kerma rate (isocenter)	Input/output characteristic
Contrast (monthly)	Mean pixel value	
Spatial resolution (monthly)	Modulation transfer function	Noise equivalent quanta
Uniformity and noise (monthly)	Noise power spectrum	

This cross‐reference show the correspondence between parts of QA procedures for image quality listed in AAPM TG‐142 planner kV imaging and image quality indicators.

According to AAPM TG‐135, there are currently no published data on tracking algorithm accuracy as a function of imager parameters such as signal‐to‐noise ratio, contrast‐to‐noise ratio, and relative MTF. Specific recommendations for the type of imager testing and expected results are thus still premature, and more work is required to establish reliable QA threshold recommendations for these tests.^18^ It was similar to AAPM TG‐142 at the point of tolerance of image quality indicators that repeat measurement and baseline comparison are required. Regarding the aspects of planner kV imaging not related to RTRT systems, a report is available concerning QA of onboard imager (OBI).^19^ In that report, resolution was evaluated on the basis of visibility (highest lp/mm allowing macroscopic check). For OBI which is primarily used for the purpose of patient setup, such visibility is important and an appropriate method of evaluation. Since in the real‐time tumor tracking system, pattern matching is performed based on pixel values and used for coordinate calculation, QA using objective indicators is effective in terms of the simplicity of the procedure, ease in control, and high precision.

As a summary of this study, we propose indicators for QA at two aspects of a diagnosis imaging device and a radiation treatment device in the RTRT system. However, because those indicators are affected by the interindividual difference of fluoroscopic devices, we cannot recommended the acceptable range, as indicated as the “tolerance level” or “action level” in the AAPM TG‐142.^4^ At some facilities, a 1.5‐mm gold marker is inserted into some sites, in addition to the 2.0‐mm gold marker, and it is necessary to verifying the relationship between ∫NEQ and *PRS* for each size of the gold markers differing in the pattern match template.^20^ Some facilities use a Visicoil as a fiducial marker in a planner kV imaging. If marker templates of the Visicoil for several X‐ray incident angles are generated from a breath‐hold computed tomography (CT), pattern matching will be improved, and the misregistration can be reduced in kV images with sufficient image quality.^21^ Some facilities use markerless tracking with a pre‐acquired image using pattern matching.^22^, ^23^ In the case of pattern matching with X‐ray images, pre‐acquired images of multiple respiratory phases are used as multiple reference templates.^22^ In the case of pattern matching with planning four‐dimensional computed tomography (4DCT) images, digitally reconstructed radiographs from one phase of a planning 4DCT are used as reference template for kV images.^23^ Therefore, we propose to use those QA procedures and indicators for another planner kV imaging system using pattern matching regardless of the type of marker used and with or without markers. Recently, new technologies for tracking of respiratory motion using positron emission tomography (PET) and magnetic resonance imaging (MRI) has also been reported.^24^, ^25^ However, in terms of versatility and popularity, tracking technologies using kV planner imaging will likely be the mainstream for the time being in radiotherapy. Especially in planner kV imaging systems using tracking with pattern matching, those QA indicators are well worth considering for assuring accuracy of 3D tracking.

## 5 CONCLUSIONS

In this study, image quality indicators for the fluoroscopic images of the RTRT system were evaluated with simplified procedures. The relationship between ∫NEQ and the gold marker 3D tracking accuracy was clarified through analysis of *PRS*. The study revealed that QA with the use of indicators, such as input/output characteristics, MTF, NPS, and ∫NEQ, was appropriate for covering all of the AAPM TG‐142 planner kV imaging items, assuring the tracking accuracy of this system. In conclusion, we proposed that those image quality indicators should be added for robust QA of the RTRT system.

## AUTHOR CONTRIBUTIONS

S.K. and M.I. wrote the main manuscript text. N.M., K.S., and R.S. gave advice on the experiment part, and H.S. is a supervisor of S.K. All authors reviewed the manuscript.

## CONFLICT OF INTEREST

No conflicts of interest.
